# 

**Published:** 1886-01

**Authors:** 


### ANNOUNCEMENT.

With this number, the JOURNAL passes into the hands of a new firm, the Journal of Health Publishing Co.

For thirty two years this publication has appeared regularly, never having missed a number.

It has become the best known Health Journal in America, both on account of its age and its general excellence.

The former editor, Wm. E. Gibbs, and the publisher, R. A. Bigelow, while severing their connection with the JOURNAL, wish to those into whose hands it has fallen, every success, and to them extend their hearty good will.

The new manager has had long experience, and we feel that under his care the publication will be made better and more satisfactory to its readers.

A competent staff of contributors has been secured, so that the articles which appear in its pages will be entirely modern and up to the times in the strictest sense of the word.

